# Introductory Lecture, Delivered at the Opening of the New Orleans School of Medicine, on the 17th Nov. 1856

**Published:** 1857-02

**Authors:** 


### BOOK NOTICES.

*Introductory Lecture, delivered at the Opening of the New Orleans School of Medicine, on the* 17*th November*, 1856. By E. D. FENNER, M.D. and Prof. of Theory and Practice of Medicine.

This is an interesting historical lecture, which will well pay for a careful perusal.

